# Hinge/floating craniotomy as an alternative technique for cerebral decompression: a scoping review

**DOI:** 10.1007/s10143-019-01180-7

**Published:** 2019-11-11

**Authors:** Hugo Layard Horsfall, Midhun Mohan, B. Indira Devi, Amos O. Adeleye, Dhaval P. Shukla, Dhananjaya Bhat, Mukhtar Khan, David J. Clark, Aswin Chari, Franco Servadei, Tariq Khan, Andres M. Rubiano, Peter J. Hutchinson, Angelos G. Kolias

**Affiliations:** 1 Division of Neurosurgery, Department of Clinical Neurosciences, Addenbrooke’s Hospital and University of Cambridge, Cambridge, UK; 2 NIHR Global Health Research Group on Neurotrauma, University of Cambridge, Cambridge, UK; 3 Department of Neurosurgery, National Institute for Mental Health and Neurosciences, Bangalore, India; 4 Division of Neurological Surgery, Department of Surgery, College of Medicine, University of Ibadan, Ibadan, Nigeria; 5 Department of Neurological Surgery, University College Hospital, Ibadan, Nigeria; 6 Department of Neurosurgery, North West General Hospital and Research Center, Peshawar, Pakistan; 7 Department of Neurosurgery, Great Ormond Street Hospital, London, UK; 8 Institute of Child Health, University College London, London, UK; 9 Department of Neurosurgery, Humanitas University and Research Hospital, Milan, Italy; 10 INUB/MEDITECH Research Group, El Bosque University, Bogota, Colombia; 11 MEDITECH Foundation, Clinical Research, Cali, Colombia

**Keywords:** Neurosurgery, Decompressive craniectomy, Traumatic brain injury, Stroke

## Abstract

Hinge craniotomy (HC) is a technique that allows for a degree of decompression whilst retaining the bone flap in situ, in a ‘floating’ or ‘hinged’ fashion. This provides expansion potential for ensuing cerebral oedema whilst obviating the need for cranioplasty in the future. The exact indications, technique and outcomes of this procedure have yet to be determined, but it is likely that HC provides an alternative technique to decompressive craniectomy (DC) in certain contexts. The primary objective was to collate and describe the current evidence base for HC, including perioperative parameters, functional outcomes and complications. The secondary objective was to identify current nomenclature, operative technique and operative decision-making. A scoping review was performed in accordance with the PRISMA-ScR Checklist. Fifteen studies totalling 283 patients (mean age 45.1 and M:F 199:46) were included. There were 12 different terms for HC. The survival rate of the cohort was 74.6% (*n* = 211). Nine patients (3.2%) required subsequent formal DC. Six studies compared HC to DC following traumatic brain injury (TBI) and stroke, finding at least equivalent control of intracranial pressure (ICP). These studies also reported reduced rates of complications, including infection, in HC compared to DC. We have described the current evidence base of HC. There is no evidence of substantially worse outcomes compared to DC, although no randomised trials were identified. Eventually, a randomised trial will be useful to determine if HC should be offered as first-line treatment when indicated.

## Introduction

A recent study estimated 60% of global neurosurgical caseload is traumatic brain injury (TBI) and stroke (6.2 and 2.8 million, respectively)—the majority in low-to-middle-income settings [1]. There are significant societal costs associated with TBI due to high levels of mortality and morbidity. A rigorous evidence base to guide treatment strategies remains an international public health priority.

The literature describing decompressive craniectomy (DC) is varied [2]. Recent and ongoing randomised controlled trials (RCTs) for the use of DC in TBI (DECRA [3]; RESCUEicp [4]; RESCUE-ASDH) and stroke (DECIMAL [5]; DESTINY [6]; HAMLET [7]) though have demonstrated its potential utility and efficacy, they raise ongoing concerns, in some of the studies, regarding the higher rates of disability observed in survivors following DC. Regarding TBI, the DECRA trial showed that neuroprotective bifrontal DC for moderate intracranial hypertension (ICP) is not helpful, whereas the RESCUEicp trial found that last-tier DC for severe and refractory ICP can significantly reduce the mortality rate but is associated with a higher rate of disability [2–4]. In relation to ischaemic stroke, a Cochrane review [8] including data from all the three extant randomised controlled trials (DECIMAL [5]; DESTINY [6]; HAMLET [7]) suggested that DC improves survival compared with best medical management, but that an increased proportion of individuals treated with DC survive with moderately severe or severe disability [8, 9].

A relatively novel and less well-utilised technique to achieve cerebral decompression in patients with brain swelling and/or raised ICP is the ‘hinge craniotomy’ (HC), also known as hinged decompressive craniectomy. The technique was first described by three independent groups in 2007 [10–12] specifically for surgical modulation of post-traumatic medically intractable raised ICP, although it has been used by neurosurgeons for several years for sundry other indications. Adoption of HC into neurosurgical practice can potentially yield benefits over traditional DC in specific situations, such as the potential to control at least moderate cerebral oedema whilst simultaneously obviating the need for a subsequent costly operative cranioplasty [2]. This is a particularly important consideration in resource-limited settings. Furthermore, unlike for the traditional surgical technique of DC, following HC, there are reports of potential reduction in axonal stretching and there are supposedly fewer complications such as syndrome of the trephined, problems with CSF hydrodynamics, infection and resorption of the autologous bone flap [13].

However, HC has possible limitations; these revolve mainly around whether sufficient extracranial brain expansion volume will be achieved and whether the patient will require the more traditional DC later on. Central to the HC vs DC debate is not just about post-operative patient survival, but the subsequent functional outcome and associated morbidity that may be incurred. There is a paucity of rigorous data evaluating HC, and contemporary evidence is based upon experience from small series in single centres from disparate regions of the world.

Our primary objective in this study was to collate, assess and describe the current evidence base for the use of HC. We assess the current indications, differing techniques, functional outcomes and complications of the procedure. To this end, we performed a scoping review; a relatively novel study design that determines the scope or coverage of a body of literature on a given topic and gives a broad overview [14, 15]. This review process is particularly useful for examining the emerging evidence relating to HC whilst it still remains relatively unclear what other, more specific questions can be posed and valuably addressed by a more precise systematic review and meta-analysis [14, 16]. This is important as there is variation in the definition and technique of HC as currently described by diverse workers interested in it from different regions of the world. Moreover, the exact indication for HC is unclear, for, although it may have a role between medical management and DC in both TBI and stroke, little robust evidence presently exists for it. A scoping review provides the perfect medium to report on the current evidence surrounding HC, using systematic methodology provided by the recently published PRISMA-ScR framework [17].

## Methods

### Protocol and registration

This scoping review has been reported in accordance with the Preferred Reporting Items for Systematic Reviews and Meta-Analysis extension for Scoping Reviews (PRISMA-ScR) [17]. Unlike systematic reviews, the protocol does not need to be registered with PROSPERO [15].

### Eligibility criteria

The following advanced search strategy was used to search all PubMed on 22 June 2019:

((((((((craniotomy[Title/Abstract]) OR craniectomy[Title/Abstract]) OR decompress*[Title/Abstract])) AND ((((((hinge*[Title/Abstract]) OR float*[Title/Abstract]) OR in situ[Title/Abstract]) OR riding[Title/Abstract]) OR osteoplastic[Title/Abstract]) OR anchored[Title/Abstract]))) NOT “case reports”[Publication Type]))

Titles and abstracts were screened for relevance. Full-text articles were then assessed for eligibility according to the PICOS criteria below. The reference lists of eligible studies and relevant articles were searched for further studies not identified by the initial search strategy. Manuscripts were excluded if data was not available separately for the HC cohort; they were case reports; or were paediatric series.

### PICOS criteria

- Population: Diagnosis of TBI or stroke and exposure to HC
- Intervention: Hinge craniotomy; in situ hinge craniectomy; the Tucci flap; in situ resin floating cranioplasty; in situ free floating craniectomy; osteoplastic decompression; hinge decompressive craniotomy temporalis; riding craniotomy; modified temporal muscle hinge decompressive craniotomy; floating anchored craniotomy
- Comparison: Studies with and without controls were included due to nature of scoping review
- Outcomes and other data collected: Demographics, description of indications and surgical techniques, intracranial pressure monitoring, mean length of stay, functional outcome, mortality
- Study design: All prospective and retrospective case series, cohort studies, case-control and randomised controlled trials with *n* > 1 written in English were included

### Selection of sources of evidence

The resulting titles and abstracts were screened independently by two authors (HLH and MM) using the PICOS criteria above. If disagreements occurred, a third author (AK) was consulted. Data extraction was performed independently by the same two authors with disagreements resolved via further review and discussion. Due to the heterogeneity of terminology used for hinge craniotomy in the global literature, whenever a new term was identified from the references, it was incorporated into the search strategy.

### Data charting process

The data extraction process in a scoping review is known as ‘data charting’. Key variables were screened and extracted from the papers. These data were inputted into a Microsoft Excel Document, which was the basis of the data charting form. This was continuously updated in an iterative process, as heterogeneity of data and reported outcomes meant often non-contiguous data points. However, if the data was important and remained an essential component to report on in this paper, it was added to the charting process. Two reviewers independently (HLH & MM) charted data from each eligible article. Any disagreements were resolved through discussion between the two reviewers or further adjudication by a third reviewer (AK).

### Data items

Indication for hinge craniotomy: age, sex and indication for hinge craniotomy. If there were multiple pathologies, for example subdural haematoma and intracerebral haemorrhage following TBI, then the first pathology was listed as the indication. For some of the studies, separate male:female ratio of HC cohort was not stated; therefore, the male:female ratio reported in Results is less than the total number of patients.

Surgical technique and nomenclature: the named procedure was recorded, with size of craniotomy, dural manipulation, hinge craniotomy practice and the placement of wound drain.

Perioperative parameters: pre- and post-operative variables (ICP, GCS, midline shift and CT-Rotterdam criteria) were recorded.

Clinical outcomes: survival, Glasgow Outcome Scale (GOS) and modified Rankin Score (mRS) and follow-up were recorded. The GOS is reported as favourable (GOS 4–5) and unfavourable (GOS 1–3). The mRS is reported as: ‘Good’ = 0–2; ‘Moderate’ = 3–4; and ‘Poor’ = 5–6.

Complications: inadequate cerebral decompression was reported if the bone flap needed to be removed subsequently due to refractory intracranial hypertension. Infection, need for reoperation and other complications were also recorded.

Comparison between HC and DC: studies that compared the two techniques were summarised.

Level of evidence: data was also extracted on article demographics (journal, institution, level of evidence, summary of article) and the income status of the country of origin.

### Synthesis of results

The results in this manuscript are presented as a scoping review, including summary tables, and follow the following format: (1) indication for hinge craniotomy; (2) surgical technique and nomenclature; (3) perioperative parameters; (4) functional outcomes; (5) complications; (6) summary of comparison studies and (7) level of evidence.

## Results

### Indication and patient demographics

**Fig. 1:**
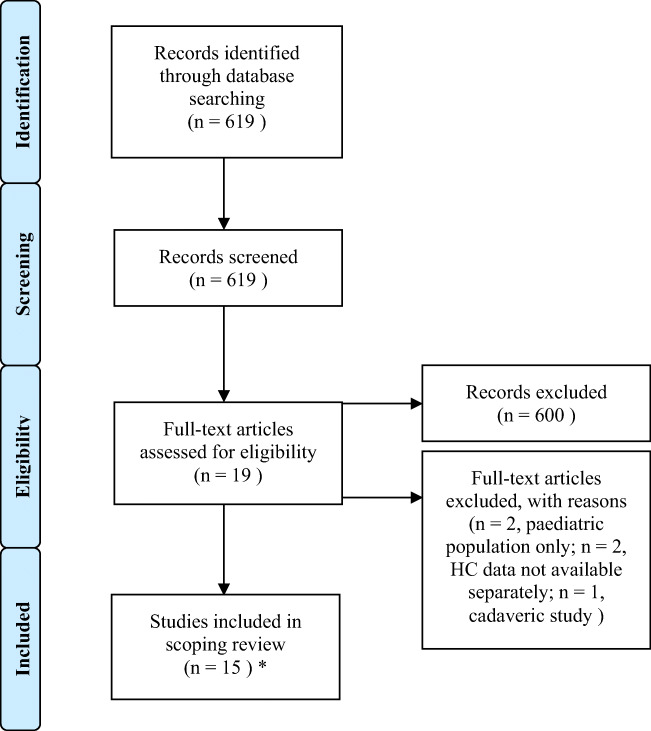
PRISMA 2009 flow diagram

A total of 15 studies [10–13, 18–28] were eligible for inclusion (Fig. 1), comprising 283 patients with a mean age 45.1 years and a male:female of 199:46 (Table 1). The majority of patients (*n* = 230, 81.3%) underwent HC following TBI. Of the patients who suffered TBI, the most common pathology was acute subdural haematoma (*n* = 182, 79.1%), followed by intracerebral haemorrhage (*n* = 33, 14.3%) and epidural haematoma (*n* = 7, 3.0%). A number of patients (*n* = 53, 18.7%) underwent HC following stroke: haemorrhagic (*n* = 40, 75.5%) and ischaemic (*n* = 13, *n* = 24.5%).

**Table 1:** Indication and patient demographics

Reference	Total patients	Pathology						Mean Age (years)	Gender (M:F)
		TBI				Stroke			
		Subdural haematoma	Epidural haematoma	Intracerebral heamatoma	Diffuse injury	Infarct	Haemorrhage		
Schmidt 2007 [11]	25	25						38.2	22:3
Ko 2007 [10]	16	5	1			3	7	51	5:11
Goettler 2007 [12]	3	2			1			–	–
Ahn 2009 [18]	7	2	1			3	1	52.7	5:2
Kenning 2009 [19]	20	11		1		4	4	50.5	14:6
Valenca 2010 [20]	4				2	1	1	44.8	1:3
Mracek 2011 [21]	20	13	1	3		2	1	42	17:3
Adeleye 2011 [22]	4	3	1					36.5	3:1
Kenning 2012 [24]	9					9		58.3	5:4
Kano 2012 [23]	21	7					14	57.4	16:5
Mezue 2013 [27]	30	5	2	17	6			36.0	–
Peethambaran 2015 [28]	10	7					3	42.7	8:2
Tsermoulas 2016 [25]	17	17						46	15:2
Adeleye 2016 [26]	40	28		12				38.4	38:2
Gutman 2017 [13]	57	57						37.2	51:5
Total	283	182	6	33	9	13	40	45.1	199:46

### Surgical technique and nomenclature

There were 12 separate terms used to describe hinge craniotomy, of which hinge craniotomy was the most common (*n* = 4, 33.3%). Every other term was used once (Table 2).

**Table 2:** Summary of surgical technique and nomenclature

Reference	Technique	Craniotomy size	Dura		Stabilisation of flap	Wound drain
			Duraplasty	Material		
Schmidt 2007 [11]	Hinge craniotomy	> 12 cm	Simple onlay	Gelfoam or Duragen	3 miniplates were fastened: a Y-shaped plate just posterior to the coronal suture, and two 2-hole plates, 1 at the sphenoid wing and a second in the posterior temporal region both below the temporalis muscle and fascia. The Y-shaped plate was secured to the surrounding skull whilst the 2-hole plates acted as buttress plates to prevent future settling.	No
Ko 2007 [10]	In situ hinge craniectomy	“Standard craniotomy”	Simple onlay	Duragen	Four miniplates. Superior plates left unfastened and inferior plates act as hinge. Hinge was refastened in 8 patients 1–2 months later. Ventriculostomy in 7 patients	No
Goettler 2007 [12]	Tucci flap	“Very large”	“Duroplasty”	Not stated	Anterior plate screwed, posterior plate not screwed	No
Ahn 2009 [18]	In situ floating resin cranioplasty	“Large”	Not stated	Not stated	Resin implant modelled intraoperatively with diameter > 5 cm larger than bone flap larger and loosely fixed with silk suture	No
Kenning 2009 [19]	Hinge craniotomy	> 12 cm	Simple onlay	Duragen	3 miniplates were fastened: a Y-shaped plate just posterior to the coronal suture, and two 2-hole plates, 1 at the sphenoid wing and a second in the posterior temporal region both below the temporalis muscle and fascia. The Y-shaped plate was secured to the surrounding skull whilst the 2-hole plates acted as buttress plates to prevent future settling	No
Valenca 2010 [20]	In-window craniotomy	12–15 cm, rectangular	Duraplasty “anteroposterior bridge between the dural edges”	Synthetic graft; homologous pericranium, fascia lata or temporal fascia	Rectangular craniotomy and subtemporal decompression. Bone flap vertically cut in two creating ‘window’. Outer frontal and parieto-occipital sides of the flap are tied to the skull at 2 points using a synthetic nonabsorbable suture to function as hinge joint, allowing opening of the window but prevents downward movement	Yes
Mracek 2011 [21]	Osteoplastic decompression	“Large hemispheral fronto-temporo-parieto-occipital flap”	Yes	Flap of pericranium or temporalis fascia	Far-near-near-far suture of the temporal muscle and depression prevented by oblique bone incision via Gigli saw	No
Adeleye 2011 [22]	Hinge DC temporalis	“Trauma bone flap”	Simple onlay— “loose expansile”	Not stated	Bone flap in situ with ipsilateral temporalis muscle; anterior and posterior vertical cuts in muscle sutured allowing mobility	Yes
Kenning 2012 [24]	Hinge craniotomy	As Kenning 2009				
Kano 2012 [23]	Hinge craniotomy	“Large”	“Duroplasty”	GORE-TEX	Additional craniectomy in the temporal squama was performed in many cases to decompress the midbrain. Miniplate to prevent flap resorption. Refastening of bone flap in 16/21 cases under local anaethesia	No
Mezue 2013 [27]	Decompressive craniotomy	“Large temporo-parietal trauma flap”	“Loosely repaired”	Autologous material, temporalis muscle or pericranium	In situ free floating or loosely sutured craniotomy	No
Peethambaran 2015 [28]	Four-quadrant osteoplastic decompressive craniectomy	“Traditional craniectomy”	Duroplasty	Synthetic patch	Bone flap divided into four-quadrants then the periosteum on each bone piece was sutured loosely to other pieces, as well as to the periosteum on one side of the calvarium with prolene/silk sutures	No
Tsermoulas 2016 [25]	Riding craniotomy	“Trauma craniotomy and wide exposure”	“Dura left open”	Not stated	Miniplates to prevent flap resorption	No
Adeleye 2016 [26]	Modified temporal muscle hDC	At least 14 cm	Duraplasty	Composite subgaleal fascia-pericranium flap	Bone flap in situ with ipsilateral temporalis muscle; anterior and posterior vertical cuts in muscle sutured allowing mobility	Yes
Gutman 2017 [13]	Floating anchored craniotomy	> 12 × 15 cm	Simple onlay	Geloforam or dural substitute	Loose vicryl sutures (1–2 cm slack) and plates (unscrewed) to prevent flap resorption and skin flap 10 cm clearance to facilitate expansion	Subgaleal

The ‘hinge’ was achieved via a variety of techniques (Table 2). Most commonly, the free bone flap was secured with miniplates. To achieve this, the inferior edge of the bone flap was re-secured to the inferior cranial edge with a straight titanium plate. The 5- or 6-millimetre screw on the inferior cranial edge protruded 1 to 1.5 mm above the plate to allow flap movement. A similar plate was screwed flush onto the superior edge of the bone flap. The distal end of the straight plate was not screwed to the nearby cranium thereby remaining unattached, and thus creating a hinge; this permitting portion of the flap to float on its hinge outward with brain swelling [11] whilst simultaneously reducing the risk of a sinking bone flap. Some of the groups fastened the upper initially unsecured screws several months later to secure the bone flap. Another technique used loosely tied sutures attached to the free bone [13] flap or a resin mould of the skull implanted [18]. This promoted symmetrical room for expansion if the brain was to swell, whilst the hinge technique provides only unilateral, and therefore asymmetrical, expansion potential. To increase expansion potential, the inner table of the free bone flap can be thinned. A separate technique, known as osteoplastic decompression [21], which has recently been adapted to the modified temporalis muscle hinge decompressive craniectomy [26], utilised the temporalis muscle as an anchor for the free bone flap which was partially secured using sutures in the anterior and posterior vertical cuts. Four reports using these techniques included the use of a subgaleal wound drain at wound closure.

### Perioperative parameters

The majority of patients undergoing HC were comatose (GCS < 9), with abnormal CT findings (significant midline shift and Rotterdam score) (Table 3). Pre-operative ICP values were sparse. Hinge craniotomy resulted in reduced ICP, a reduction in midline shift and also improved Rotterdam scores (Table 3). The GCS was not reported as a post-operative outcome. Specifically, of the studies that had pre-operative ICP recorded, patients undergoing HC demonstrated a reduced ICP post-operatively. Patients in the Guttman et al. [13] series had pre-operative ICP of 32.7 ± 8.1 mmHg compared to 16.0 ± 12.1 mmHg post-operatively. In Valenca et al. [20], the pre-operative ICP range was 15–35 mmHg and 6–12 mmHg post-operatively.

**Table 3:** Perioperative data

Reference	Pre-operative				Post-operative		
	ICP (mmHg)	GCS	MLS (mm)	Rotterdam score	ICP (mmHg)	MLS (mm)	Rotterdam score
Schmidt 2007 [11]		GCS < 9 (24/25 pt)	10.6 (13/25 pt)			5.1 (13/25 pt)	
Ko 2007 [10]		Avg GCS 6 to 7			Range 2–22 (7/16 pt)		
Goettler 2007 [12]							
Ahn 2009 [18]		8	9			6.7	
Kenning 2009 [19]		4.1 (mean motor GCS)	11.0 ± 4.70	4.8 ± 1.1	12.0 ± 5.6	6.4 ± 4.4	3.2 ± 1.0
Valenca 2010 [20]	Range 15–35 (1/4 pt)						
Mracek 2011 [21]		3–8 (20/20 pt)	10		14.5	3	
Adeleye 2011 [22]		8–9 (range) (4/4 pt)					
Kenning 2012 [24]		4.7 (mean motor GCS)	8.5 ± 6.1	3.4 ± 1.3	10.8 ± 3.4	6.0 ± 3.9	2.9 ± 0.8
Kano 2012 [23]		3–6 (9/21 pt); 7–12 (11/21 pt); 13–15 (1/21 pt)			25.5 ± 17.0 (17/21 pt)		
Mezue 2013 [27]		3–8 (24/30 pt); 9–12 (6/30 pt)	> 10 (12/30 pt)				
Peethambaran 2015 [28]		7	13.1 ± 4.78			6.6 ± 3.9	
Tsermoulas 2016 [25]		3–8 (9/17 pt); 9–12 (5/17 pt); 13–15 (3/17 pt)		4	Intracranial HTN index (13.8)		
Adeleye 2016 [26]		3–8 (15/40 pt); 9–12 (17/140 pt); 13–15 (8/40 pt)	> 5 (36/40 pt)	≥ 4 (36/40 pt)			
Gutman 2017 [13]	32.7 ± 8.1	≤ 8 (32/57 pt)	7.3 ± 5.57	3.6 ± 1.2	16.0 ± 12.1	2.6 ± 3.8	

Direct comparison of HC to DC in relation to perioperative variables was limited (Table 6). Kenning et al. [19] demonstrated that ICP control at post-operative day 5 was adequate and equivalent (HC 12.1 ± 2.6 mmHg; DC 15.0 ± 6.3 mmHg), despite the smaller volume of expansion (HC 77.5 ± 54.1 ml; DC 105.1 ± 65.1 ml). The imaging in 15 of the patients revealed reversal of midline shift (MLS) pre- and post-operatively (HC post-op MLS 6.4 ± 4.4 mm; DC 5.5 ± 4.6 mm), although this difference was not statistically significant [19]. Furthermore, the MLS in patients receiving HC was also reduced. Peethambaran et al. [28] demonstrated a MLS of 13.1 ± 4.78 mm reduced to 6.6 ± 3.9 mm post-operatively, whilst Schmidt et al. [11] 10.6 to 5.1 mm following HC.

The same group 3 years later demonstrated no statistically significant difference in post-operative ICP control for the duration of monitoring (HC 10.8 ± 3.4 mmHg; DC 11.9 ± 3.5 mmHg) [24], in addition to a similar finding of a smaller expansion volume (HC 77.6 ± 44.7 ml; DC 96.3 ± 54.4 ml) [24]. Furthermore, Tsermoulas et al. [25] compared DC to ‘riding craniotomy’ (HC from hereon) in ASDH, demonstrating the post-operative intracranial hypertension index was not worse than DC (HC 13.8; DC 16.6), in a patient cohort with similar baseline characteristics.

### Clinical outcomes

Two hundred and eighty-three patients underwent HC, of which 211 survived (74.6%). There was a paucity of data reported relating to functional outcome and duration of follow-up (Table 4).

**Table 4:** Functional outcome data. GOS: ‘Good’ = 4–5; ‘Poor’ = 1–3. mRS: ‘Good’ = 0–2; ‘Moderate’ = 3–4; ‘Poor’ = 5–6

Reference	Survival	Functional outcome at discharge unless otherwise stated			Length of follow-up (months)
	*n*, % of total pt	GOS	mRS		
Schmidt 2007 [11]	13, 52%	NR	NR		NR
Ko 2007 [10]	14, 87.5%	NR	NR		10
Goettler 2007 [12]	2, 66.6%	NR	NR		NR
Ahn 2009 [18]	6, 85.7%	Good: 2 (28.6%); Poor: 5 (71.4%)	NR		NR
Kenning 2009 [19]	15, 75%	NR	NR		NR
Valenca 2010 [20]	4, 100%	NR	NR		2–14
Mracek 2011 [21]	16, 80%	Good: 8 (40%); Poor: 12 (60%)	NR		Up to 6
Adeleye 2011 [22]	4, 100%	GOSE ‘near normal’	NR		3–18
Kenning 2012 [24]	5, 56%	3.6 ± 0.6 (at 1–3 months)	2.8 ± 1.1 (at 1–3 months)		12
Kano 2012 [23]	19, 90.4%	Good: 3 (43%); Poor: 4 (57%)	Good: 1 (7%); Moderate: 9 (64%); Poor: 4 (29%)		13.7 ± 11.2 (18 cases)
Mezue 2013 [27]	24, 80%	Good: 16 (53%); Poor: 14 (47%)	NR		NR
Peethambaran 2015 [28]	3, 30%	NR	NR		6
Tsermoulas 2016 [25]	14, 82.0%	Good: 11 (64.7%); Poor: 6 (35.3%)	NR		6
Adeleye 2016 [26]	28, 70%	Good: 27 (67.5%); Poor: 13 (32.5%)	NR		11
Gutman 2017 [13]	44, 77.2%	NR	Pre-discharge: Good: 31 (54.3%); Moderate: 10 (17.5%); Poor: 14 (24.6%); NA: 2 (3.5%)	Post-discharge: Good: 22 (38.6%); Moderate: 3 (5.3%); Poor: 13 (22.8%); NA: 10 (17.5%)	NR

GOS: ‘Good’ = 4–5; ‘Poor’ = 1–3. mRS: ‘Good’ = 0–2; ‘Moderate’ = 3–4; ‘Poor’ = 5–6. NR = not reported by authors.

Comparing survival outcomes of HC to DC, Kenning et al. [19] found no significant difference between hospital survival (HC *n* = 15, 75%; DC *n* = 21, 70%), whilst their second study investigating HC in stroke management found that hospital survival was significantly higher in the DC group (HC *n* = 5, 56%; DC *n* = 17, 89%, *p* = 0.04) [24] (Table 6). However, they also found that despite the higher in-hospital mortality, HC was associated with better long-term functional outcome, as determined by mRS scores at the 30–90 day period (HC 2.8 ± 1.1; DC 4.4 ± 0.9, *p* = 0.01) and at the 90- to 180-day period (HC 2.5 ± 0.6; DC 3.9 ± 1.0, *p* = 0.03) [24]. A study from India [28] also demonstrated that both DC and ‘four-quadrant osteoplastic decompressive craniotomy’ (hinge craniotomy in this case) were comparable in relation to the duration of surgery, duration of ICU stay and survival (*p* > 0.05). Furthermore, there was significant brain expansion potential and reversal of MLS. Tsermoulas et al. [25] also found that more patients in the DC group had poor functional status at 6 months compared with the HC group at 6 months (HC *n* = 6, 35%; DC *n* = 41, 59%). On the contrary, Kano et al. [23] compared DC with hinge craniotomy following TBI or stroke and found no significant difference in the long-term functional outcome, as measured by post-operative GOS and mRS. There were variable lengths of follow-up, if at all stated. Nine studies explicitly stated the length of follow-up, which ranged from 2 to 18 months. Furthermore, not all studies recorded functional outcome status at the end of follow-up, merely just that the patient was seen by healthcare practitioners in the time period. Six studies did not state duration of follow-up.

### Complications

There were 54 reported complications in the HC cohort (Table 5). Nine patients (*n* = 9/283, 3.2%) required subsequent decompressive craniectomy due to uncontrollable ICP, or in other words, ‘failure’ of hinge craniotomy. In one study [23], the bone flap was removed in 2 cases due to acute hydrocephalus or brain herniation causing low cerebral perfusion pressure and in the other 2 cases, ICP was elevated immediately secondary to CT-confirmed epidural haemotoma requiring progression to DC. In another study [24], one patient was placed in a barbiturate-induced coma at the request of the treating neurologist, although the paper states that ICP was in normal range.

**Table 5:** Complications of hinge craniotomy and decompressive craniectomy if available comparative study

Reference	Hinge craniotomy					Decompressive craniectomy		
	Number of patients	Progression to DC	Infection	Cranioplasty	Other	Number of patients	Complications excluding infection	Infection
Schmidt 2007 [11]	25	0	1	1 (due to wound infection)	Other infection (6); seizure (1)	NA: No craniectomy group		
Ko 2007 [10]	16	0	0	0	Subgaleal collection—resolved (3)			
Goettler 2007 [12]	3	0	0	0				
Ahn 2009 [18]	7	0	0	1 (patient requested; cosmetic)				
Kenning 2009 [19]	20	0	0	0	Reoperation—indication not stated (3)	30	Reoperation—indication not stated (3)	
Valenca 2010 [20]	4	0	0	0		NA: No craniectomy group		
Mracek 2011 [21]	20	2	0	0	Removal of bone flap—malfunction of technique (2)			
Adeleye 2011 [22]	4	0	0	0				
Kenning 2012 [24]	9	0	1	1	Reoperation to secure mobile bone plate (1); haematoma progression (1); subdural effusion (1); barbiturate-induced coma (1)	19	Subdural effusion (11); evolution of contralateral mass lesions (1); hydrocephalus (2); reoperation—cranioplasty (17); reoperation—not cranioplasty (7); contusion progression (11)	Infection (5)
Kano 2012 [23]	21	4	0	0		37		Bone flap infection (6)
Mezue 2013 [27]	30	0	2	0		8		Infection—meningitis (1)
Peethambaran 2015 [28]	10	1	2	0		10	Hydrocephalus (4)	Infection (3);
Tsermoulas 2016 [25]	17	0	0	0	Reoperation – indication not stated (4)	69	Reoperation (41: excluding cranioplasty, including CSF diversion, evacuation of post-operative heamatoma, burr holes for subdural collection, surgical debridement for infection, lobectomy)*	Infection*
Adeleye 2016 [26]	40	0	4	0	Bone flap sinking (2); hydrocephalus (1); other infection (4)	NA: No craniectomy group		
Gutman 2017 [13]	57	2	2	0	EVD insertion (2); subgaleal haematoma (1)			
Total	283	9	12	3	30	173	97	15

*No breakdown of complications provided

Regarding the replaced free bone flap specifically, there were 2 incidences of malfunctioning technique requiring removal of the bone flap [22] and two incidences of bone flap depression [26] utilising the temporalis HC technique. One patient required reoperation to secure the bone flap due to increased mobility [19]. There was no reported syndrome of the trephined or other complications uniquely associated with decompressive craniectomy. In the reported studies, one patient, who underwent HC with a resin implant, requested subsequent cranioplasty for cosmetic reasons [18]. The remaining patients receiving HC group had satisfactory cosmetic outcomes. Ko et al. [10] refastened the hinge in 8 patients during the proceeding post-operative months, using local anaesthetic. Two patients refused this and subsequently died within the 8 months of follow-up.

Kenning et al. [19, 24] found that there was no significant difference between HC and DC in terms of operative time, need for reoperation, duration of mechanical ventilation or ICU stay. Their analysis revealed a greater degree (not statistically significant) of post-operative parenchymal contusion enlargement with DC, which may reflect blossoming of the contusions secondary to unconstrained brain expansion [19]. Furthermore, Kenning et al. reported only one patient undergoing HC requiring subsequent cranioplasty compared to 17 patients who received DC (HC 1/5, 20%; DC 17/17, 100%) [24].

Kano et al. [23] report no bone flap infections in HC whilst there were 6 of such in DC after autologous cranioplasty (*p* = 0.02). The earliest of the six cases of bone flap infection in DC occurred 1 week after the cranioplasty, and the latest case occurred more than 4 months after cranioplasty (mean, 4.1 weeks). Additionally, we extracted data of the complications from the DC groups of the controlled studies. There were 15 infections (8.7%) in the DC group versus 12 infections (4.2%) in the HC (*p* = 0.065, Fisher’s).

### Level of evidence

The level of evidence for the majority of the HC literature was poor, according to the University of Oxford’s Centre for Evidence-based Medicine Levels of Evidence [29]. There was 1 level V study [12] (6.7%), 8 level IV studies [10, 11, 13, 18, 20–22, 26] (53.3%) and 6 level III studies [19, 22, 24, 25, 27, 28] (40.0%). Twelve studies were retrospective in nature. Five studies [10–12, 19, 24] were from the USA, the most common origin of study. Four studies were from LMIC countries [22, 26–28]—India and Nigeria. The remaining 11 studies were in developed healthcare settings.

## Discussion

This review highlights the heterogeneity of nomenclature as well as the technical variations in the operative procedure of HC as currently reported in the global literature. Nonetheless, the evidence base, still limited though, regarding its indications and effectiveness suggests that HC may have a role to play in the treatment of TBI and/or stroke, obviating secondary complications usually associated with decompressive craniectomy, as well as the cost and complications of the consequent cranioplasty.

### Nomenclature and technique

Since being described by three groups in 2007 [10–12], HC has 12 different terms to our knowledge. The most common term was ‘hinge craniotomy’ (Table 2). Despite heterogenous techniques and nomenclature, the general principles appear unanimous: partial decompression to relieve raised intracranial pressure with subsequent immediate replacement of the bone flap, over the surgical cranial window, in a ‘loose’ or ‘hinged’ fashion in an attempt to accommodate cerebral oedema and subsequent swelling, without further neurosurgical intervention. This principal attempts to reduce complications such as infection, syndrome of the trephined, hydrodynamic disturbances and secondary cranioplasty whilst maintaining adequate cerebral decompression.

**Fig. 2:**
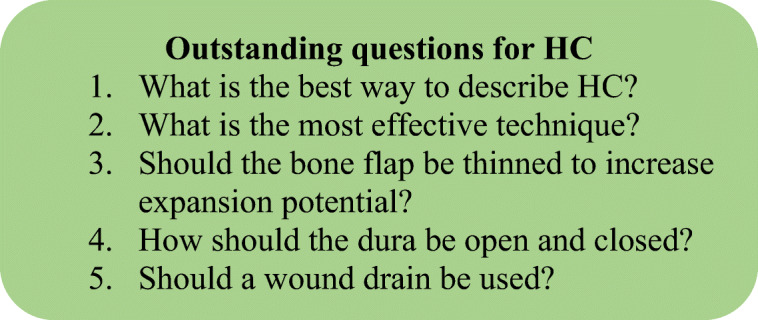
Outstanding questions for HC

We believe that from hereon, it would be useful to agree and adhere to one umbrella term (e.g. decompressive craniotomy), and this can be used to describe all variations of this technique. Nevertheless, the following questions (Fig. 2) remain open to the neurosurgical community.

### Efficacy of hinge craniotomy

#### Intracranial pressure

Hinge craniotomy was reported to be effective at controlling ICP. All studies that reported post-operative readings demonstrated a decrease in ICP and associated reduction in midline shift (Table 3). The studies comparing HC to DC suggest that HC is at least as effective as DC in this respect (Table 6). Kano et al. [23] compared HC with DC in TBI and stroke, and summarising their data, averred ‘the hinge technique with ICP monitoring was effective and safe for the management for head trauma and stroke’. Furthermore, Kenning et al. [19, 24] suggested that ‘hinge craniotomy was at least as good as decompressive craniectomy in providing post-operative ICP control’. Furthermore, out of 283 patients undergoing HC, only 9 patients (3.2%) required subsequent DC, suggesting that adequate cerebral decompression was achieved by HC alone. In the RESCUEicp trial, which enrolled patients with severe TBI and ICP raised and refractory to medical management, of the patients who were randomised and received a DC (*n* = 187), 12 required a barbiturate infusion post-DC due to ongoing issues with ICP control (6.4%). Obviously, the patients included in the studies of the present scoping review are not directly comparable to the RESCUEicp population but this figure can give an indication as to the proportion of patients with refractory ICP despite DC.

**Table 6:** Summary table of studies comparing HC to DC

Reference	Pt (*n*)	Indication				Outcomes									
		TBI		Stroke		ICP (mmHg)		Mean MLS (mm)		GOS		mRS		Survival *n* (%)	
		HC	DC	HC	DC	HC	DC	HC	DC	HC	DC	HC	DC	HC	DC
Kenning 2009 [19]	50	12	18	8	12	12.1 ± 2.6	15.0 ± 6.3	6.4 ± 4.4	5.5 ± 4.6	Not stated				15 (75)	21 (70)
Kenning 2012 [24]	28	–		9	19	10.8 ± 3.4	11.9 ± 3.5	6.0 ± 3.9	5.3 ± 5.4	NA		2.8 ± 1.1 at 30–90 days post-op	4.4 ± 0.9 at 30–90 days post-op	5 (56)	17 (89)
Kano 2012 [23]	58	7	19	14	18	25.5 ± 17.0	Not reported			Good: 3 (50%); Poor: 3 (50%)	Good: 4 (21%); Poor: 15 (79%)	Good: 1 (7%); Moderate: 9 (64%); Poor: 4 (29%)	Good: 1 (6%); Moderate: 6 (33%); Poor: 11 (61%)	19 (94)	*
Mezue 2013 [27]	38	30	8	–		Not reported				Good: 16 (53%); Poor: 14 (47%)	Good: 2 (25%); Poor: 6 (75%)			24 (80)	6 (75)
Peethambaran 2015 [28]	20	10	9		1	Not reported		6.6 ± 3.9	6.0 ± 2.1	Not stated				3 (30)	5 (50)
Tsermoulas 2016 [25]	86	17	69	–		13.8^	16.6^	Not reported		Good: 11 (64.7%); Poor: 6 (35.3%)	Good: 28 (40.1%); Poor: 41 (59.9%)	NA		14 (82)	43 (62)

^Expressed as intracranial hypertension index

*Unable to extract mortality as not explicitly stated, only grouped as mRS 5–6

This concept is further reinforced by a biomechanical study in human cadaver skulls [30], which compared DC to HC and ‘dynamic decompressive craniotomy’ and the effect on ICP after abrupt increase in intracranial volume. They found that both the dynamic craniotomy and the HC techniques provided significant control of ICP during 120 ml increase in intracranial volume as compared with craniotomy rigidly fixed with plates (4.86 mmHg, 8.36 mmHg, 44.84 mmHg, respectively).

#### Clinical and functional outcomes

Central to the clinical management of raised ICP in TBI or stroke is the patient’s quality of life after receiving treatment for the injury. This was highlighted in the recent RESCUEicp trial [4] which demonstrated that more patients with refractory ICP (> 25 mmHg) who underwent decompressive craniectomy had decreased mortality but increased disability when compared with medical therapy alone. In RESCUEicp [4], the survival of the DC patients at 6 months was 73.1%. The overall survival of patients undergoing HC was 74.6% (*n* = 211) (Table 4) and thus very similar to the RESCUEicp mortality, although it is important to appreciate that RESCUEicp was looking at secondary DC. The studies included in this manuscript had limited long-term functional outcome reported (Tables 4 and 6). Furthermore, the presence of extracranial injuries or injuries of different severity makes direct summary of outcomes difficult. Lastly, as the evidence informing treatment strategies in TBI is developing, morbidity outcome post-surgical intervention is key and thus central to optimal intervention.

#### Complications

The standard procedure of DC is associated with several complications, including syndrome of the trephined, subdural hygromas/effusions, contusion/haematoma progression, incisional cerebrospinal fluid leak and hydrocephalus. In contrast, HC might provide a means to reduce these complications, particularly obviating the need for subsequent costly cranioplasty, further hospital admission and likely prolonged ITU stay. Nevertheless, one quite significant complication of the latter to consider is the possibility of ‘failure’ of HC to provide adequate ICP control and therefore progression to DC, of which there were 6 cases in this review (Table 5). Otherwise, the data presented in this study suggest that HC is associated with a trend towards reduced infection and complications described above (Table 5). Due to the retrospective nature of the vast majority of studies, underreporting of complications may be a potential issue and obviously due to the lack of randomisation, differences in the baseline characteristics could be responsible for the observed trends.

### Hinge craniotomy in low-to-middle-income settings

Perhaps one of the most interesting facets to this review is the emerging practice during HC of using the temporalis muscle, without any other costly biomaterial implant, as an anchor for the bone flap in low-to-middle-income settings. Adeleye et al. [22, 26] report on this technique (Table 2), as well as a low-cost duraplasty, and using a unitized tube-and-reservoir urine drainage system, the Uri-bag, as a wound drain to significantly reduce the cost of TBI treatment. Obviating the need for cranioplasty further reduces costs and logistical issues. This is particularly relevant as the LMIC have the majority of TBI burden, with the least resources, facilities and trained neurosurgeons [1, 31]. Therefore, hinge craniotomy provides potential clinical utility, in addition to economic benefits, further reinforcing the need for a more substantial evidence base relating to its use in these settings.

### Novel devices

Central to developing this field of neurosurgery are innovative devices to facilitate surgical theory. Two devices, the ‘Skull Flap’ (SF) [32] and the ‘expandable dynamic craniotomy bone flap fixation plate’ [33], provide biomechanical evidence of utility in controlling ICP by providing adequate volume expansion whilst preventing sinking of the bone flap. Furthermore, the devices are durable, low-cost and easy to use.

The SF [32] is a hinge system comprised with plate and sliding track that carries a locking-unlocking system, connected to a titanium wire tunnelled and externalised in the scalp. This wire serves as traction for repositioning of the flap back to its anatomical position once cerebral oedema has subsided, permitting bony fusion of the flap edges.

Another recent manuscript explores the biomechanics of a ‘novel expandable dynamic craniotomy bone flap fixation plate’ [33]. The dynamic plate comprises solid ends with holes for placement of screws into the bone flap at one end and the skull at the other end. The solid portions are connected with a configuration of flat interconnections that function similarly to a spring that reversibly expands and contracts as well as angulates, depending upon the tension exerted. The plates allow outward bone flap movement to accommodate an increase in ICP and/or intracranial volume and retract the bone flap in a flush position once the ICP normalizes. The group evaluated the plates characteristics in human cadaver skulls and demonstrated significant increase in intracranial volume expansion due to compliance of the bone flap in comparison to a rigid or hinged bone flap. In essence, the reversibly expandable, MRI/CT-compatible plates provide for a low-profile bone flap fixation with rigid restriction of bone flap sinking and also enable cranial decompression with a high tolerance for repetitive expansion and contraction.

### Cost-effectiveness

An important aspect to consider especially for low- and middle-income countries is if HC is actually cost-effective or at least cost neutral compared to DC. Unfortunately, raw cost data was not available in the included studies. However, DC is thought to be an expensive procedure with a mean patient cost of over US$ 20,000 when performed in a developing country [34]. HC is potentially a cheaper alternative due to autologous tissues being utilised and reducing the need for a second operation for a cranioplasty [26]. A formal cost-effectiveness analysis evaluating the many different factors that influence cost needs to be undertaken, which is one of the future aims of our neurotrauma study group at the University of Cambridge.

### Limitations of the present study

The lack of robust comparative HC vs DC data, the combined indications (TBI and stroke) for HC in some of the reports, heterogenous pre-operative and radiological metrics, paucity of pre-operative ICP monitoring, short-term follow-up and poor reporting of long-term functional outcomes makes absolute conclusion difficult. Furthermore, GOS recorded at discharge is not necessarily a true representative of long-term outcomes, and the paucity of robust follow-up investigating associated morbidity, a key metric, in most reports is also of note. Additionally, mRS is the most commonly used outcome scale in the field of stroke but many of the included studies used the GOS to assess outcomes in the TBI but also stroke patients. The two scales examine slightly different aspects of functional outcome but the Glasgow Outcome Scale correlates well with the mRS in patients with stroke [35].

### Developing the evidence base

To continue developing the evidence base for HC, we would advocate following the IDEAL Methodology [36]. This is a 5-stage description of the surgical development process, a crucial tool for systematic evaluation of surgical innovation and that is instrumental for achieving improved design, conduct and reporting of surgical research. Currently, HC is between stage IIb and III ‘Exploration’ and ‘Assessment’, i.e. the technique is stable, has been replicated by numerous study groups and there is some literature demonstrating comparison to existing practice (DC).

It must be appreciated that HC cannot, as yet, be considered an alternative to all the DCs, but rather an alternative to primary DC, not to secondary DC. Whereas HC has been performed most often for persistent brain swelling after evacuation of intracranial hematoma, DC has been performed most often as a part of second- or third-tier therapy as in DECRA [3] and RESCUEicp [4] trials for patients with medically refractory intracranial hypertension. In such cases, it is unlikely that a HC will be performed instead of DC.

To further develop HC, additional evaluation of the technique prospectively and co-operatively may help mature consensus over definition, quality and indications. Ultimately, an international effort, with a multi-centre randomised controlled trial, with participation from low- and middle-income countries is required. The trial could compare HC to DC with criteria for progression from HC to DC in selected cases. In addition, ICP monitoring, if already used clinically, would aid meaningful comparison. Importantly, such a study would aim to compare long-term functional outcomes and surgical morbidity (Table 7).

**Table 7:** Summary of evidence. Level of evidence as per Oxford Centre for Evidence Medicine 2009^29^

Reference	Journal	Institution	Economic status	Type of study	Level of evidence	Summary
Schmidt 2007 [11]	Journal of Neurosurgery	West Virginia University Health Sciences Center Charleston Division, USA	HIC	Retrospective case series	IV	Technical description of HC; HC provided adequate cerebral decompression I small sample; no complications usually associated with DC.
Ko 2007 [10]	Operative Neurosurgery	Weill Cornell Medical College, USA	HIC	Retrospective case series	IV	Technical description of HC; HC provided adequate cerebral decompression in small sample; no complications usually associated with DC.
Goettler 2007 [12]	Journal of Trauma-Injury Infection & Critical Care.	Brody School of Medicine, USA	HIC	Small case series	V	Technical description of TF; provides less decompression volume than DC but was adequate; suggested reduced morbidity vs DC.
Ahn 2009 [18]	Journal of Korean Neurosurgical Society	Wonkwang University School of Medicine, Korea	HIC	Retrospective case series	IV	Technical description; perhaps ISRFC better able to accommodate cerebral odema than HC; obviates need for cranioplasty; particularly useful in elderly population.
Kenning 2009 [19]	Neurosurgical Focus	Albany Medical Centre, USA	HIC	Retrospective case control	IIIb	Compared ICP outcomes between HC and DC: HC appears to be at least as good as DC in providing post-operative ICP control and results in equivalent early clinical outcomes.
Valenca 2010 [20]	Journal of Neurosurgery	Federal University of Pernambuco, Brazil	UMIC	Retrospective case series	IV	‘In-window’ craniotomy is an alternative solution for deploying autologous material and obviates need for secondary surgery.
Mracek 2011 [21]	Acta Neurochirurgica	Charles University Hospital and Faculty of Medicine in Pilsen, Czech Republic	HIC	Retrospective case series	IV	ODC is effective at reducing ICP in a subgroup of patients where DC would be too radical; obviates need for reoperation and associated complications of DC.
Adeleye 2011 [22]	Surgical Neurology International	University College Hospital, Ibadan, Nigeria	LMIC	Retrospective case series	IV	Effective cerebral decompression using autologous tissue in LMIC setting
Kenning 2012 [24]	Journal of Neurosurgery	Thomas Jefferson University Hospital, USA	HIC	Retrospective case control	IIIa	HC appears to be at least as good as DC in providing post-operative ICP control at a similar therapeutic index; in-hospital mortality was higher in HC patients but superior long-term functional outcomes; HC may help limit post-operative complications.
Kano 2012 [23]	Neurologia Medico-Chirurgic	Fukaya Red Cross Hospital, Japan	HIC	Prospective cohort study	IIIa	HC with ICP monitoring was effective and safe for head trauma or stroke; not associated with bone flap infection; follow up of 13–40 months.
Mezue 2013 [27]	Nigerian Journal of Clinical Practice	University of Nigeria Teaching Hospital, Nigeria	LMIC	Retrospective cohort study	IIIa	HC sufficient and able to control ICP in selected TBI cases. Severe head injury still requires DC but associated with increased mortality.
Peethambaran 2015 [28]	Neurology India	Government Medical College, India	LMIC	Prospective observational case control	IIIb	Technique provides adequate and comparable decompression to DC
Tsermoulas 2016 [25]	World Neurosurgery	Queen Elizabeth Hospital, Birmingham, UK	HIC	Retrospective observational cohort study	IIIb	Compared reoperations, functional outcome and ICP in DC, RC and FC; DC was not associated with better outcomes; replace flap if conditions allow
Adeleye 2016 [26]	Journal of Neurological Surgery Part A	University College Hospital, Ibadan, Nigeria	LMIC	Prospective cohort study	IV	Further development of temporalis hinge DC technique in LMIC setting; good outcomes in mild to moderate TBI
Gutman 2017 [13]	Surgical Neurology International	Gold Coast University Hospital, Australia	HIC	Retrospective case series	IV	FAC provides symmetrical decompression vs HC; FAC provided adequate cerebral decompression

*HIC* high-income country, *UMIC* upper-middle-income country, *LMIC* low-to-middle-income country, *LIC* low-income country

## Conclusion

Hinge craniotomy has a potential role in the surgical management of TBI/stroke, yielding adequate cerebral decompression in the majority of reported cases, a reduction in complications and potentially offers substantial economic savings (both operative costs and the cost of living with significant morbidity). It is likely that HC offers an intermediate intervention between treatment-refractive medical therapy and traditional decompressive craniectomy. Future work should aim to facilitate a global consensus about HC and its utility as treatment, ultimately paving the way for a randomised controlled trial.

## References

[1] Dewan, M. C. et al. (2018) Estimating the global incidence of traumatic brain injury. J Neurosurg 1–18. 10.3171/2017.10.jns17352 10.3171/2017.10.JNS17352 29701556

[2] Kolias AG (2018). The current status of decompressive craniectomy in traumatic brain injury. Curr Trauma Rep.

[3] Cooper JD (2011). Decompressive craniectomy in diffuse traumatic brain injury. New Engl J Med.

[4] Hutchinson PJ (2016). Trial of decompressive craniectomy for traumatic intracranial hypertension. New Engl J Med.

[5] Vahedi K (2007). Sequential-design, multicenter, randomized, controlled trial of early decompressive craniectomy in malignant middle cerebral artery infarction (DECIMAL Trial). Stroke.

[6] Jüttler E (2007). Decompressive surgery for the treatment of malignant infarction of the middle cerebral artery (DESTINY). Stroke.

[7] Hofmeijer J (2009). Surgical decompression for space-occupying cerebral infarction (the Hemicraniectomy After Middle Cerebral Artery infarction with Life-threatening Edema Trial [HAMLET]): a multicentre, open, randomised trial. Lancet Neurol.

[8] Cruz-Flores S, Berge E, Whittle IR (2012) Surgical decompression for cerebral oedema in acute ischaemic stroke. *Cochrane Db Syst Rev*. 10.1002/14651858.cd003435.pub2 10.1002/14651858.CD003435.pub2 PMC11491187 22258954

[9] Kolias AG, Kirkpatrick PJ, Hutchinson PJ (2013). Decompressive craniectomy: past, present and future. Nat Rev Neurol.

[10] Ko K, Segan S (2007). In situ hinge craniectomy. Oper Neurosurg.

[11] Schmidt JH, Reyes BJ, Fischer R, Flaherty SK (2007). Use of hinge craniotomy for cerebral decompression. J Neurosurg.

[12] Goettler CE, Tucci KA (2007). Decreasing the morbidity of decompressive craniectomy: the Tucci Flap. J Trauma Acute Care.

[13] Gutman M, How E, Withers T (2017). The floating anchored craniotomy. Surg Neurol Int.

[14] Armstrong R, Hall BJ, Doyle J, Waters E (2011). ‘Scoping the scope’ of a cochrane review. J Public Health.

[15] Munn Z et al (2018) Systematic review or scoping review? Guidance for authors when choosing between a systematic or scoping review approach. *BMC Med Res Methodol* **18**(143) 10.1186/s12874-018-0611-x PMC6245623 30453902

[16] Munn Z, Stern C, Aromataris E, Lockwood C, Jordan Z (2018) What kind of systematic review should I conduct? A proposed typology and guidance for systematic reviewers in the medical and health sciences. *BMC Med Res Methodol* **18**(5) 10.1186/s12874-017-0468-4 PMC5761190 29316881

[17] Tricco AC et al (2018) PRISMA extension for Scoping Reviews (PRISMA-ScR): checklist and explanation. *Ann Intern Med*. 10.7326/m18-0850 10.7326/M18-0850 30178033

[18] Ahn D-H, Kim D-W, Kang S-D (2009). In situ floating resin cranioplasty for cerebral decompression. J Korean Neurosurg S.

[19] Kenning TJ, Gandhi RH, German JW (2009). A comparison of hinge craniotomy and decompressive craniectomy for the treatment of malignant intracranial hypertension: early clinical and radiographic analysis. Neurosurg Focus.

[20] Valença MM, Martins C, da Silva J (2010). “In-window” craniotomy and “bridgelike” duraplasty: an alternative to decompressive hemicraniectomy. J Neurosurg.

[21] Mracek J, Choc M, Mork J, Vacek P, Mracek Z (2011). Osteoplastic decompressive craniotomy—an alternative to decompressive craniectomy. Acta Neurochir.

[22] Adeleye AO, Azeez A (2011). Decompressive craniectomy bone flap hinged on the temporalis muscle: a new inexpensive use for an old neurosurgical technique. Surg Neurol Int.

[23] Kano T, Kurosaki S, Wada H (2012). Retrospective analysis of hinge technique for head trauma or stroke. Neurol Med-Chir.

[24] Kenning TJ (2012). Cranial decompression for the treatment of malignant intracranial hypertension after ischemic cerebral infarction: decompressive craniectomy and hinge craniotomy. J Neurosurg.

[25] Tsermoulas G (2016). Surgery for acute subdural hematoma: replace or remove the bone flap?. World Neurosurg.

[26] Adeleye AO (2016). Clinical and radiologic outcome of a less invasive, low-cost surgical technique of osteoplastic decompressive craniectomy. J Neurol Surg Part Central European Neurosurg.

[27] Mezue W, Ndubuisi C, Ohaegbulam S, Chikani M, Erechukwu U (2013). Cranial bony decompressions in the management of head injuries: decompressive craniotomy or craniectomy?. Niger J Clin Pract.

[28] (2015) Four-quadrant osteoplastic decompressive craniotomy: a novel technique for refractory intracranial hypertension - A pilot study. Neurol India 63**:**895–902 10.4103/0028-3886.170081 26588623

[29] (2009) Oxford Centre for Evidence-based Medicine – Levels of Evidence (March 2009)

[30] Khanna R, Ferrara L (2016). Dynamic telescopic craniotomy: a cadaveric study of a novel device and technique. J Neurosurg.

[31] Dewan MC, et al. (2018) Global neurosurgery: the current capacity and deficit in the provision of essential neurosurgical care. Executive Summary of the Global Neurosurgery Initiative at the Program in Global Surgery and Social Change. J Neurosurg 1–10. 10.3171/2017.11.jns171500 10.3171/2017.11.JNS171500 29701548

[32] Chibbaro S (2013). The ‘Skull Flap’ a new conceived device for decompressive craniectomy/cranioplasty: feasibility study on cadaver specimen. J Neurosci Rural Pract.

[33] Khanna R, Ferrara L, Khanna S (2019) Biomechanics of a novel reversibly expandable dynamic craniotomy bone flap fixation plate. *J Neurosurg*:1–8. 10.3171/2018.8.jns172614 10.3171/2018.8.JNS172614 30611148

[34] Badke G (2018). Analysis of direct costs of decompressive craniectomy in victims of traumatic brain injury. Arq Neuropsiquiatr.

[35] Kasner SE (2006). Clinical interpretation and use of stroke scales. Lancet Neurol.

[36] McCulloch P (2009). No surgical innovation without evaluation: the IDEAL recommendations. Lancet.

